# Multicenter cohort association study of *SLC2A1* single nucleotide polymorphisms and age-related macular degeneration

**Published:** 2012-03-17

**Authors:** Dominique C. Baas, Lintje Ho, Michael W.T. Tanck, Lars G. Fritsche, Joanna E. Merriam, Ruben van het Slot, Bobby P.C. Koeleman, Theo G.M.F. Gorgels, Cornelia M. van Duijn, André G. Uitterlinden, Paulus T.V.M. de Jong, Albert Hofman, Jacoline B. ten Brink, Johannes R. Vingerling, Caroline C.W. Klaver, Michael Dean, Bernhard H. F. Weber, Rando Allikmets, Gregory S. Hageman, Arthur A.B. Bergen

**Affiliations:** 1 Department of Clinical and Molecular Ophthalmogenetics, The Netherlands Institute for Neuroscience (NIN), an institute of the Royal Netherlands Academy of Arts and Sciences (KNAW), Amsterdam, The Netherlands; 2 Department of Epidemiology, Erasmus Medical Center (EMC), Rotterdam, The Netherlands; 3 Department of Ophthalmology, EMC, Rotterdam, The Netherlands; 4 Department of Clinical Epidemiology, Biostatistics & Bioinformatics, Academic Medical Center (AMC), Amsterdam, The Netherlands; 5 Institute of Human Genetics, University of Regensburg, Regensburg, Germany; 6 Department of Ophthalmology, Pathology and Cell Biology, Columbia University, New York, NY; 7 Department of Medical Genetics Research Section, Utrecht Medical Center (UMC), Utrecht, The Netherlands; 8 Department of Internal Medicine, EMC, Rotterdam, The Netherlands; 9 Department of Ophthalmology, AMC, Amsterdam, The Netherlands; 10 Laboratory of Experimental Immunology, Cancer and Inflammation Program, National Cancer Institute, Frederick, MD; 11 Department of Ophthalmology and Visual Sciences, The University of Iowa, IA; 12 Center for the Study of Macular Degeneration, University of California, Santa Barbara, CA; 13 Department of Clinical Genetics, AMC, Amsterdam, The Netherlands

## Abstract

**Purpose:**

Age-related macular degeneration (AMD) is a major cause of blindness in older adults and has a genetically complex background. This study examines the potential association between single nucleotide polymorphisms (SNPs) in the glucose transporter 1 (*SLC2A1*) gene and AMD. *SLC2A1* regulates the bioavailability of glucose in the retinal pigment epithelium (RPE), which might influence oxidative stress–mediated AMD pathology.

**Methods:**

Twenty-two SNPs spanning the *SLC2A1* gene were genotyped in 375 cases and 199 controls from an initial discovery cohort (the Amsterdam-Rotterdam-Netherlands study). Replication testing was performed in The Rotterdam Study (the Netherlands) and study populations from Würzburg (Germany), the Age Related Eye Disease Study (AREDS; United States), Columbia University (United States), and Iowa University (United States). Subsequently, a meta-analysis of SNP association was performed.

**Results:**

In the discovery cohort, significant genotypic association between three SNPs (rs3754219, rs4660687, and rs841853) and AMD was found. Replication in five large independent (Caucasian) cohorts (4,860 cases and 4,004 controls) did not yield consistent association results. The genotype frequencies for these SNPs were significantly different for the controls and/or cases among the six individual populations. Meta-analysis revealed significant heterogeneity of effect between the studies.

**Conclusions:**

No overall association between *SLC2A1* SNPs and AMD was demonstrated. Since the genotype frequencies for the three *SLC2A1* SNPs were significantly different for the controls and/or cases between the six cohorts, this study corroborates previous evidence that population dependent genetic risk heterogeneity in AMD exists.

## Introduction

Age-related macular degeneration (AMD) is the most common cause of severe visual impairment in Western countries, rendering the disease a major public health issue [1,2].

The prevalence of AMD increases strongly with age, affecting 4% of the population over the age of 60 and more than 10% of individuals older than 75 [2,3]. The early stages of the disease are characterized by drusen, focal depositions of extracellular material in Bruch’s membrane beneath the retinal pigment epithelium (RPE) [4,5]. Late stages of the disease include two forms: an atrophic form (geographic atrophy [GA]) and an exudative form (choroidal neovascularization [CNV]).

AMD has a multifactorial etiology [6]. Age, smoking history, high body mass index, hypertension, and hypercholesterolemia influence AMD predisposition [7]. The importance of genetic risk factors for AMD was highlighted in several recent studies. In addition to the complement factor H (*CFH*) gene, genetic association studies consistently implicated at least four complement genes (factors B [*CFB*] and I [*CFI*], components 2 [*C2*] and 3 [*C3*]) as well as one of two genes (*ARMS2* and *HTRA1)* in the chromosomal region 10q26. Taken together, these data suggest that the complement system, oxidative stress, mitochondrial function, and extracellular matrix turnover play a role in AMD [8–14].

The RPE is one of the key tissues involved in AMD and functions in several processes that are vital for preserving sight. The RPE layer constitutes the outer blood-retinal barrier and regulates transport ions, fluid, and metabolites between the retina and the choroid [15]. Among other things, the RPE transports glucose to the photoreceptors. Glucose supply to the photoreceptors is essential since glucose is the preferred energy substrate for the metabolically highly active retina [16]. The sodium-independent glucose transporter *SLC2A1* is the predominant glucose transporter in the retina [17,18]. *SLC2A1* localizes to the apical and basolateral membranes of the RPE [19]. According to Beatty and colleagues, the retina is the ideal environment for generating free radicals and other reactive oxygen species [20]. This may occur through similar transport mechanisms through the inner and outer blood retina barriers, and makes the retina an environment susceptible to oxidative damage. Fernandes et al. showed in 2011 that sustained oxidative stress can result in decreased glucose transport in retinal endothelial cells [21]. On the other hand, increased glucose transport and *SLC2A1* expression are upregulated by hypoxia as shown by Takagi et al. [22]. Finally, increased serum glucose (hyperglycemia) might lead to impaired antioxidant protection [23] and increased reactive oxygen species (ROS) production [24]. In conclusion, DNA sequence variations or altered expression levels in *SLC2A1* may influence glucose delivery to the retina and thereby profoundly affect local oxidative stress.

Variants in *SLC2A1* have been associated with diabetic retinopathy [25], type 2 (non-insulin-dependent) diabetes [26], diabetic nephropathy [27,28], and clear-cell renal cell carcinoma [29]. Finally, expression of *SLC2A1* in the retina and brain is altered in different pathophysiological conditions, including hypoxia [22,30], Alzheimer disease [31], and epilepsy [32]. We hypothesized that genetic variants in *SLC2A1* could influence the glucose transport capabilities of this transporter. This would lead to changes in the glucose level in the RPE and neural retina, and alter the local oxidative burden. Since multiple studies suggest that oxidative stress is implicated in AMD [20,33–35], we performed an extensive case-control association analysis, to test whether *SLC2A1* gene variants are associated with this devastating disorder.

## Methods

### Study populations

We employed five case-control studies and one prospective cohort study, consisting of a total of 5,235 AMD cases and 4,203 ethnically- and age-matched control subjects. The studies were approved by the Ethics Committees of the Academic Medical Center Amsterdam, the Erasmus Medical Center Rotterdam, the University of Würzburg, the Age Related Eye Disease Study (AREDS) Access Committee, and the Institutional Review Boards of Columbia University, and the University of Iowa. All studies followed the tenets of the Declaration of Helsinki, and all participants provided signed informed consent.

The initial discovery sample, the Amsterdam-Rotterdam-Netherlands (AMRO-NL) study population, consisted of 375 unrelated individuals with AMD and 199 control individuals. Subjects were all Caucasian and recruited from the Erasmus University Medical Centre, Rotterdam, and the Netherlands Institute for Neuroscience, Amsterdam, the Netherlands [36].

The second sample, the Rotterdam Study, is a prospective cohort study aimed at investigating chronic diseases in older adults, as previously described [37]. The eligible population comprised all inhabitants aged 55 years or older of a middle-class suburb in Rotterdam, the Netherlands.

The third sample, the Franconian AMD study (Würzburg, Germany), consisted of 612 cases and 794 age-matched control subjects; the cases and controls originated from the lower Franconian region of Bavaria, Germany [38].

The fourth sample, the AREDS (United States), included two parts: 1) a randomized clinical trial to assess the effect of supplemental antioxidants on risk of AMD and cataract, which began in April 1992 and ended in November 2001; and 2) a longitudinal study of progression of AMD. The study participants were Caucasian, aged 55 to 80 years, and were recruited at 11 centers in the United States from clinic and general populations in those areas. The sample consisted of 936 individuals with AMD and 218 controls [39].

The fifth sample, the Columbia University (United States) study population, comprised 1,104 unrelated individuals with AMD and 368 unrelated controls of European American descent, recruited at Columbia University [40].

The sixth sample, the Iowa University (United States) study population, comprised 1,139 unrelated individuals with AMD and 403 unrelated controls of European American descent, recruited at the University of Iowa [41].

### Diagnosis of age-related macular degeneration

Subjects from all cohorts underwent ophthalmic examination and fundus photography as described [36–41]. Signs of AMD were graded according to the International Classification and Grading system for AMD [42] except the AREDS [43] and the Franconian AMD study [38] (Appendix 1).

### Single nucleotide polymorphism selection

Twenty-two SNPs were selected that capture common variations in the *SLC2A1* gene. SNP data were from the Centre d’Étude du Polymorphisme Humain (CEPH) population (Utah residents with ancestry from northern and western Europe) by use of the International HapMap Project. SNP selection was based on criteria such as functional relevance, minor allele frequency (MAF)>10%, coverage of the main linkage disequilibrium (LD) blocks, and tagging of the most common haplotypes. Tag SNPs were selected with Tagger, an option of Haploview [44] (all SNPs were captured with an LD tagging criterion of *r^2^*>0.8).

### Genotyping

Genomic DNA was isolated from peripheral leukocytes after venous puncture according to standard protocols. A total of 1,536 SNPS, including 22 *SLC2A1* SNPs, were genotyped in the AMRO-NL study population using an Illumina GoldenGate assay on a BeadStation 500 GX (Illumina Inc., San Diego, CA). This GoldenGate assay did not include our previous published complement factor 5 (*C5*) and *ERCC6* versus AMD screenings. We currently screened all known rare and common genetic variants, which met Illumina quality standards, in approximately 45 AMD candidate genes (DB and AB; data not shown). The Rotterdam Study was genotyped with the Illumina HumanHap 550K array (Illumina Inc.). Quality control was performed using PLINK (version 1.01) [37]. The Franconian AMD study sample was genotyped with TaqMan SNP Genotyping assay for the SNPs rs4660687: A>C and rs3754219: A>C. Rs841853 was genotyped with PCR (forward primer: 5′-CCT CAG GGA ATA AAG CTA GTC TCC AG-3′; reverse primer: 5′-AGA CCA GCC AGA GGT TCC AAA-3′) followed by XbaI digestion and restriction fragment length analysis. The AREDS, Columbia, and Iowa samples were also genotyped with Taqman SNP genotyping assays. All TaqMan assays were performed on ABI7300 Real-Time PCR systems (Applied Biosystems, Foster City, CA).

### Statistical analysis

To account for multiple comparisons, we determined, initially, three different significance tresholds for the Golden-Gate Assay in the AMRO-NL study. The first was an overall Bonferroni corrected significance threshold per SNP (0.05/ total nr of SNPs on the Illumina GoldenGate assay [n=1536; p=3.25 × 10^−5^]). However, this significance level takes neither the large difference in the MAF of the SNPs screened nor the co-occurrence of SNP variation in LD blocks into account. Thus, next to this overall significance level, we also applied a gene-wise significance threshold by dividing the nominal alpha by the number of SNPs per gene. The gene-wise significance threshold for *SLC2A1* is 0.05/ total nr of *SLC2A1* SNPs on the Illumina GoldenGate assay (n=22; p=2.27 × 10^−3^). Finally, as a positive control to determine the true statistical significance levels of our assay, we also included complement factor 3 (*C3*) polymorphisms, well known in the literature to associate with AMD. We found an association with multiple *C3* SNPs and AMD in our study, with p values ranging from p=0.02 to p=0.0003 (not shown). The p values of our initial *SLC2A1* association findings ranged from p=0.02 to p=0.0006 (see the Results section).

In all studies, we employed the χ^2^ test to test SNP distributions for conformity with the Hardy–Weinberg equilibrium (HWE). We compared allele frequencies between cases and controls using Fisher’s exact test statistic. We estimated odds ratios (ORs) and 95% confidence intervals (CIs) for the risk of AMD with logistic regression analysis and represent the risk of disease (AMD versus no AMD) in the genetic risk group divided by the risk of disease in the non-risk group (non-carriers). For the AMRO-NL study and the Rotterdam Study, we adjusted all ORs for age and gender. To determine whether *SLC2A1* SNPs were independent risk factors, we estimated their effects in a model with an additional adjustment for known (genetic) risk factors: *CFH* Y402H, *LOC387715* A69S, and smoking and their interactions with the *SLC2A1* SNPs. All analyses were performed in Statistical Package for Social Sciences (SPSS) for Windows software (release 16.0; IBM-SPSS, Inc., Chicago, IL). In the Rotterdam Study, we performed association tests using logistic regression in the PLINK software package (version 1.01) [45]. We performed a meta-analysis using Review Manager (RevMan) Version 5.0. assuming an additive genetic model. We tested heterogeneity between studies using Cochran’s Q statistic [46]. In addition, we quantified heterogeneity with the *I^2^* metric [46]. In the absence of heterogeneity (*I*^2^ statistic <25%), we used the fixed effects model (the Mantel-Haenszel method) to calculate the pooled OR; otherwise, we used the random effects model (the DerSimonian and Laird method). We compared genotype frequencies between the study populations with the Pearson χ^2^ test.

## Results

### The AMRO-NL sample: associations of *SLC2A1* SNPs with age-related macular degeneration

We initially genotyped 22 SNPs in 375 unrelated AMD patients and 199 controls of the AMRO-NL study population. Demographic characteristics of the AMRO-NL study population and all study populations used were essentially described previously [36,47]. SNP genotype frequencies were tested in controls and conformed to the HWE, except for two SNPs (rs12407920, rs16830101; p=0.04) that were removed from further analysis. HWE data, genotype distributions, and allelic p values for the 22 *SLC2A1* SNPs are presented in Table 1. Three out of the 22 SNPs showed a significant allelic association with AMD: rs3754219 (p=0.0011), rs4660687 (p=0.0157), and rs841853 (p=0.0006). The reported initial associations in the AMRO-NL study did not reach the overall Bonferroni significance threshold, but two out of three SNPs (rs3754219 and rs841853) passed the gene-wise threshold as well as the positive control (*C3*) association threshold. The risks of AMD for these three *SLC2A1* SNPs, adjusted for age and gender, are presented in Table 2. When all AMD cases and controls of the AMRO-NL study were included in the analysis, we observed significant association with all three SNPs. SNP (rs3754219) showed a protective effect for AMD (OR 0.44; 95% CI 0.26–0.74). The other SNPs showed a risk increasing effect (rs4660687, rs841853) with the highest ORs seen for homozygous carriers of the risk alleles of rs841853 (OR 2.24; 95% CI 1.13–4.41). We subsequently analyzed the early and late AMD subgroups separately. For early AMD, we observed a significant association only for rs4660687 (Table 2). For the late AMD subgroup, we observed a similar effect as seen for all AMD cases: a significantly increased risk for hetero- and homozygote carriers of rs841853 and for the heterozygote carriers of rs4660687 and a protective effect for homozygote carriers of rs3754219 (Table 2). Adjusting for three prominent AMD risk factors (*CFH* Y402H, *LOC38771*5 A69S, and smoking) did not modify the relation of any of the *SLC2A1* SNPs with AMD (data not shown).

**Table 1:** Allelic association between age-related macular degeneration and 22 selected *SLC2A1* single nucleotide polymorphisms in the AMRO-NL study population

**No AMD (controls)**					**AMD (cases)**					
**SNP ID**	**AA number (%)**	**Aa number (%)**	**aa number (%)**	**m.a.f**	**AA number (%)**	**Aa number (%)**	**aa number (%)**	**m.a.f**	**Allelic p-value**	**HWE**
rs1105297	94 (51.4)	75 (41.0)	14 (7.7)	28.1	171 (47.2)	159 (43.9)	32 (8.8)	30.8	0.3652	yes
rs1770810	115 (62.8)	59 (32.2)	9 (4.9)	21	232 (64.4)	117 (32.5)	11 (3.1)	19.3	0.5203	yes
rs1770811	109 (59.9)	64 (33.7)	9 (4.9)	22.5	229 (63.3)	122 (33.7)	11 (3.0)	19.9	0.3419	yes
rs12407920	161 (89.9)	16 (8.9)	2 (1.1)	5.5	293 (82.1)	64 (17.9)	0 (0.0)	9	0.0538	no
rs16830101	161 (89.9)	16 (8.9)	2 (1.1)	5.5	292 (82.0)	64 (18.0)	0 (0.0)	9	0.0509	no
rs3754219	**44 (24.6)**	**84 (46.9)**	**51 (28.5)**	**52**	**118 (33.0)**	**184 (51.4)**	**56 (15.6)**	**41.3**	**0.0011**	**yes**
rs3754223	108 (59.7)	67 (37.0)	6 (3.3)	21.8	225 (62.2)	124 (34.3)	13 (3.6)	20.7	0.6935	yes
rs3768043	108 (59.0)	68 (37.2)	7 (3.8)	22.4	219 (60.5)	129 (35.6)	14 (3.9)	21.7	0.8162	yes
rs4660687	**71 (47.3)**	**57 (38.0)**	**22 (14.7)**	**33.7**	**95 (34.1)**	**133 (47.7)**	**51 (18.3)**	**42.1**	**0.0157**	**yes**
rs4660691	109 (59.6)	68 (37.2)	6 (3.3)	21.9	224 (62.0)	124 (34.3)	13 (3.6)	20.8	0.6947	yes
rs710221	64 (35.0)	96 (52.5)	23 (12.6)	38.8	106 (29.4)	193 (53.6)	61 (16.9)	43.8	0.1193	yes
rs710222	64 (35.2)	96 (52.7)	22 (12.1)	38.5	108 (29.8)	193 (53.3)	61 (16.9)	43.5	0.1182	yes
rs751210	94 (51.4)	75 (41.0)	14 (7.7)	28.1	170 (47.2)	158 (43.9)	32 (8.9)	30.8	0.4003	yes
rs841845	114 (62.3)	60 (32.8)	9 (4.9)	21.3	231 (64.0)	119 (33.0)	11 (3.0)	19.5	0.5222	yes
rs841848	114 (62.3)	60 (32.8)	9 (4.9)	21.3	230 (63.5)	121 (33.4)	11 (3.0)	19.8	0.5767	yes
rs841851	114 (62.3)	60 (32.8)	9 (4.9)	21.3	232 (64.1)	119 (33.0)	11 (3.0)	19.5	0.4725	yes
rs841852	114 (62.3)	60 (32.8)	9 (4.9)	21.3	231 (64.0)	119 (33.0)	11 (3.0)	19.5	0.5222	yes
rs841853	**104 (58.1)**	**62 (34.6)**	**13 (7.3)**	**24.6**	**150 (41.8)**	**168 (47.0)**	**41 (11.4)**	**34.9**	**0.0006**	**yes**
rs841856	113 (61.7)	61 (33.3)	9 (4.9)	21.6	227 (63.4)	120 (33.5)	11 (3.1)	19.8	0.524	yes
rs841858	129 (70.5)	47 (25.7)	7 (3.8)	16.7	250 (69.3)	102 (28.3)	9 (2.5)	16.6	1	yes
rs900836	109 (59.6)	68 (37.2)	6 (3.3)	21.9	224 (62.0)	124 (34.3)	13 (3.6)	20.8	0.6947	yes
rs900837	109 (59.6)	68 (37.2)	6 (3.3)	21.9	224 (62.0)	124 (34.3)	13 (3.6)	20.8	0.6947	yes

AMD=age-related macular degeneration; SNP=single nucleotide polymorphism; m.a.f=minor allele frequency; HWE=Hardy–Weinberg Equilibrium. “A” indicates common allele, “a” minor allele. Genotype frequencies are given as a percentage of subjects succesfully genotyped. The allelic p-value is calculated with Fisher's exact test. The χ2 test was used to test SNP distributions for conformity with HWE.

**Table 2:** Odds Ratios (OR) and 95% confidence intervals (CI) of AMD cases versus controls of the AMRO-NL study population for three SNPs in *SLC2A1*

** **	**No AMD**	**All AMD cases**		**Early AMD**		**Late AMD**	
	n=179	n=358		n=91		n=267	
rs3754219	**No. (%)**	**No. (%)**	**OR (95%CI)**	**No. (%)**	**OR (95%CI)**	**No. (%)**	**OR (95%CI)**
**Genotype**							
Noncarrier (AA)	44 (24.6)	118 (33.0)	1	23 (25.3)	1	95 (35.6)	1
Heterozygous (Aa)	84 (46.9)	184 (51.4)	0.82 (0.53–1.27)	52 (57.1)	1.32 (0.72–2.43)	132 (49.4)	0.73 (0.46–1.14)
Homozygous (aa)	51 (28.5)	56 (28.5)	0.44 (0.26–0.74)	16 (17.6)	0.59 (0.28–1.27)	40 (15.0)	0.37 (0.21–0.66)
m.a.f (%)	51.9	41.3		46.2		39.7	
	n=150	n=279		n=82		n=197	
rs4660687	**No. (%)**	**No. (%)**	**OR (95%CI)**	**No. (%)**	**OR (95%CI)**	**No. (%)**	**OR (95%CI)**
**Genotype**							
Noncarrier (AA)	71 (47.3)	95 (34.1)	1	24 (29.3)	1	71 (36.0)	1
Heterozygous (Aa)	57 (38.0)	133 (47.7)	1.74 (1.13–2.70)	41 (50.0)	2.13 (1.15–3.93)	92 (46.7)	1.61 (1.01–2.57)
Homozygous (aa)	22 (14.7)	51 (18.3)	1.73 (0.96–3.12)	17 (20.7)	2.29 (1.04–5.01)	34 (17.3)	1.55 (0.82–2.90)
m.a.f (%)	33.7	42.1		45.7		40.6	
	n=179	n=359		n=91		n=268	
rs841853	**No. (%)**	**No. (%)**	**OR (95%CI)**	**No. (%)**	**OR (95%CI)**	**No. (%)**	**OR (95%CI)**
**Genotype**							
Noncarrier (AA)	104 (58.1)	150 (41.8)	1	47 (51.6)	1	103 (38.4)	1
Heterozygous (Aa)	62 (34.6)	168 (47.0)	1.87 (1.27–2.77)	37 (40.7)	1.44 (0.84–2.46)	131 (48.9)	2.21 (1.42–3.39)
Homozygous (aa)	13 (7.3)	41 (11.4)	2.24 (1.13–4.41)	7 (7.7)	1.32 (0.49–3.55)	34 (12.7)	2.64 (1.28–5.46)
m.a.f (%)	24.6	34.9		28		37.1	

AMD=age-related macular degeneration; m.a.f=minor allele frequency. “A” indicates common allele, “a” minor allele. Percentages not always add up to 100% because of rounding. ORs were estimated by logistic regression. Adjusted for age and sex.

### Independent replication studies have variable outcomes

**Figure 1:**
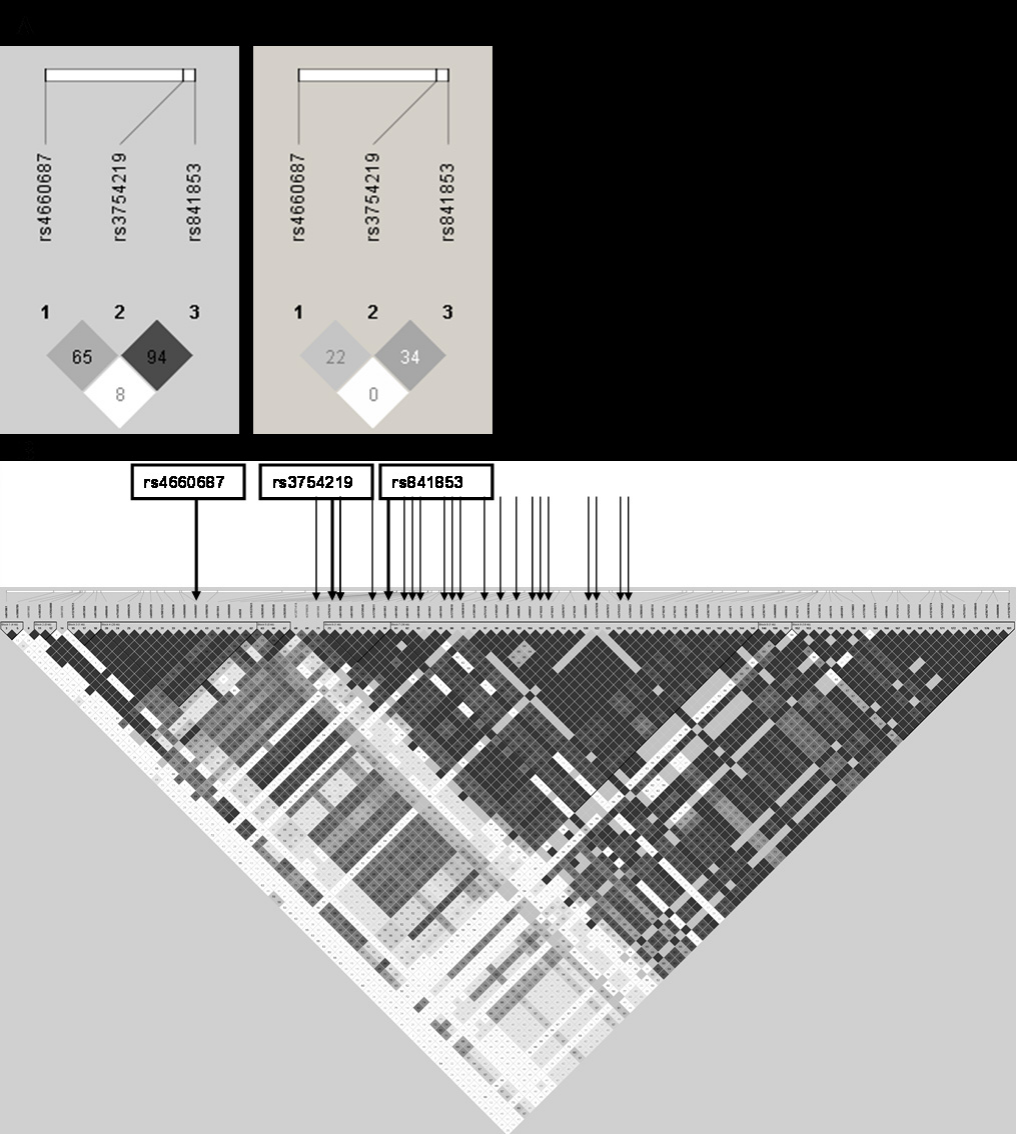
Linkage disequilibrium (LD) display in Haploview of (**A**) the three *SLC2A1* single nucleotide polymorphisms (SNPs) rs3754219, rs4660687, and rs841853. Left: D’(D prime), Right: r^2^ (r square) (**B**) all the 22 SNPs in the *SLC2A1* gene with minor allele frequency>10% screened in this study and illustrating the nine distinct haplotype blocks. LD scores: D' (D prime) displays the raw D' value, which is the normalized covariance for a given marker pair and the correlation coefficient r^2^ (r square). Darker shades represent stronger LD.

The three SNPs that showed allelic and genotypic association (rs3754219, rs4660687 and rs841853) were selected for replication in five study populations of comparable ethnic (Caucasian) composition: the Rotterdam Study, the Franconian AMD study, the AREDS, Columbia University, and the University of Iowa. Figure 1A shows the LD map in Haploview of the three SNPs and the corresponding LD scores, and Figure 1B shows the LD map of all 22 *SLC2A1* SNPs with minor allele frequency>10% screened in this study and illustrates the nine distinct haplotype blocks. The three associated SNPs are in two different blocks in the *SLC2A1* gene (rs3754219 and rs841853 are in the same LD block). As can be seen, two SNPs (rs1770811 and rs841856) without allelic association are inside the LD block with rs3754219 and rs841853. Obviously, the observed (non-) associations depend on the informativeness (MAF) of the specific SNPs and the distribution of alleles. A possible explanation might also be differences in LD patterns (between the SNPs in the same wild-type LD block) with an untyped causal variant.

### Replication studies for *SLC2A1* single nucleotide polymorphism rs3754219

The results of replicating the association between SNP rs3754219 and AMD (all cases or late AMD and controls only) are presented in Appendix 2. Genotype frequencies for rs3754219 followed the HWE in all study populations (data not shown). When we included all AMD cases, we observed no significant association with rs3754219 in any of the replication cohorts. When we analyzed the AMD subgroups separately (Appendix 3), we found a borderline significant effect in the Rotterdam Study for the CNV cases (p=0.0554). In line with the results of the discovery cohort, we observed this possible protective effect for homozygous carriers of the risk allele. In contrast, we observed a risk increasing association effect between *SLC2A1* rs3754219 and AMD (subtype) in the Columbia University study: This effect occurred in the homozygous carriers of the risk allele (OR 1.66; 95% CI 1.01–2.72) in the GA group (Appendix 3). No significant associations were seen in the other replication cohorts.

### Replication studies for *SLC2A1* single nucleotide polymorphism rs4660687

The results of replicating the association between SNP rs4660687 and AMD (all cases or late AMD and controls only) are presented in Appendix 2. Genotype frequencies followed the HWE in all study populations (data not shown). The association of rs4660687 with AMD, as found in the AMRO-NL study, was confirmed in the Rotterdam Study (OR 1.39; 95% CI 1.12–1.72). Thus, both Dutch populations showed a significant association for all AMD cases. However, in the other replication cohorts, no significant association was found. Upon subtype analysis (Appendix 4), we found a significant association between rs4660687 and the early AMD cases of the Rotterdam Study. Again, this effect was present in both Dutch populations. For this SNP, we also found a possible opposite effect: in the Franconian AMD study, we observed a significant protective effect in the mixed AMD subgroup instead of a risk increasing effect. This was seen for hetero- and homozygous carriers of the risk allele. In the three cohorts from the United States, we found no significant associations for AMD.

### Replication studies for *SLC2A1* single nucleotide polymorphism rs841853

The results of replicating the association between rs841853 SNP and AMD (all cases or late AMD and controls only) are presented in Appendix 2. Genotype frequencies followed the HWE in all study populations (data not shown). The association of rs841853 with AMD, as found in the AMRO-NL study, was confirmed in the AREDS sample: We observed a significant effect for homozygous carriers of the risk allele (OR 2.07; 95% CI 1.14–3.76) in all AMD cases. In the other replication cohorts, no significant association was found. Upon subtype analysis (Appendix 5), we observed a significant association between rs841853 and the CNV cases of the Rotterdam Study. Additionally, in the AREDS sample, we observed a significant association between rs841853 and late AMD cases (Appendix 2). The effect was particularly profound for the CNV and GA subpopulations (Appendix 5). Finally, in the Columbia cohort, we found a significant association between rs841853 and the GA cases, but this effect was in the opposite direction: heterozygotes showed a protective association instead of a risk increasing effect for GA. We also saw this protective effect for the late AMD cases of the Columbia cohort (Appendix 2). We saw no significant associations between rs841853 and AMD subtypes in the two other study populations.

### Meta-analysis: heterogeneity of effect

We performed a meta-analysis of the putative association between AMD and *SLC2A1* SNPs rs3754219, rs4660687, and rs841853 across the independent study populations.

**Figure 2:**
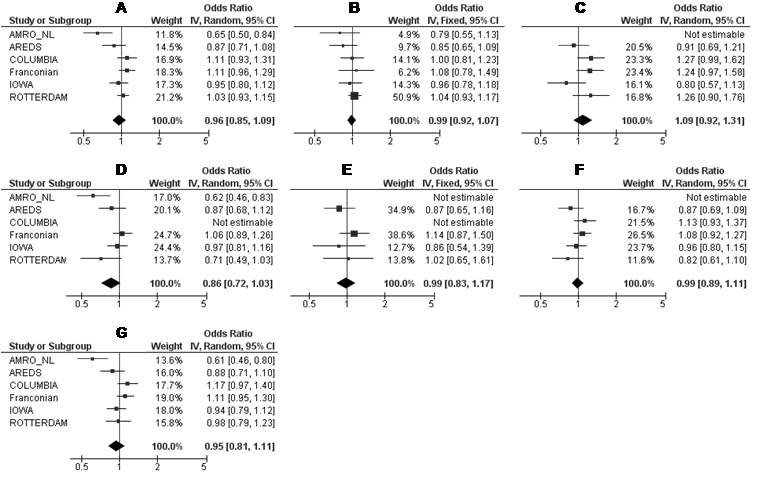
Meta-analysis of rs3754219 for its association with all AMD cases (**A**), early (**B**), GA (**C**), CNV (**D**), mixed (**E**), CNV + mixed (**F**), and late AMD (**G**) cases in six independent study populations. AMD=age-related macular degeneration; GA=geographic atrophy; CNV=choroidal neovascularization; Mixed cases (combination of GA + CNV). Odds ratios (ORs, squares) and 95% confidence intervals (CI; horizontal lines) are presented for each study. Also shown is the black diamond of the summary OR using either the additive Mantel-Haenszel fixed effects model when heterogeneity was absent (*I*^2^ <25%) or the additive DerSimonian and Laird random effects model when heterogeneity was present (*I*^2^ >25%). Heterogeneity between studies was tested using Cochran's Q statistic and the I-square statistic for inconsistency.

**Figure 3:**
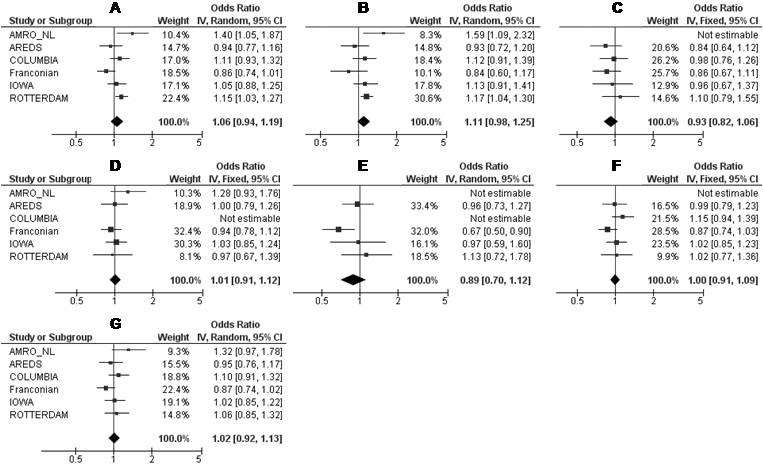
Meta-analysis of rs4660687 for its association with all AMD cases (**A**), early (**B**), GA (**C**), CNV (**D**), mixed (**E**), CNV + mixed (**F**), and late AMD (**G**) cases in six independent study populations. AMD=age-related macular degeneration; GA=geographic atrophy; CNV=choroidal neovascularization; Mixed cases (combination of GA + CNV). Odds ratios (ORs, squares) and 95% confidence intervals (CI; horizontal lines) are presented for each study. Also shown is the black diamond of the summary OR using either the additive Mantel-Haenszel fixed effects model when heterogeneity was absent (*I*^2^ <25%) or the additive DerSimonian and Laird random effects model when heterogeneity was present (*I*^2^ >25%). Heterogeneity between studies was tested using Cochran's Q statistic and the I-square statistic for inconsistency.

**Figure 4:**
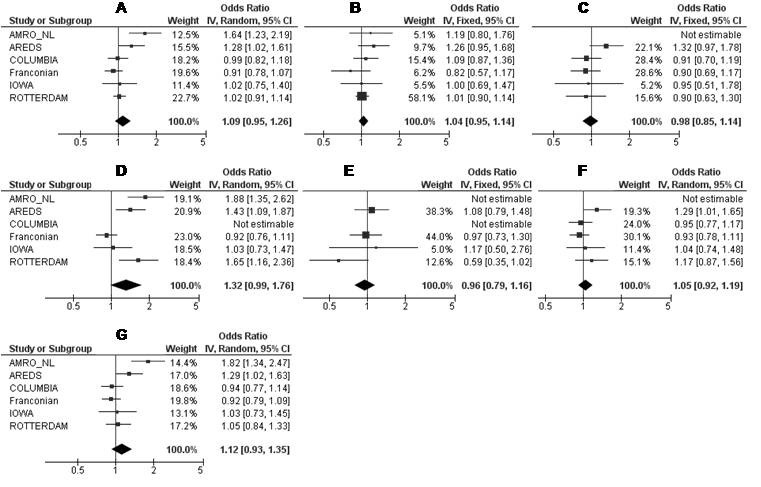
Meta-analysis of rs841853 for its association with all AMD cases (**A**), early (**B**), GA (**C**), CNV (**D**), mixed (**E**), CNV + mixed (**F**), and late AMD (**G**) cases in six independent study populations. AMD=age-related macular degeneration; GA=geographic atrophy; CNV=choroidal neovascularization; Mixed cases (combination of GA + CNV). Odds ratios (ORs, squares) and 95% confidence intervals (CI; horizontal lines) are presented for each study. Also shown is the black diamond of the summary OR using either the additive Mantel-Haenszel fixed effects model when heterogeneity was absent (*I*^2^ <25%) or the additive DerSimonian and Laird random effects model when heterogeneity was present (*I*^2^ >25%). Heterogeneity between studies was tested using Cochran's Q statistic and the I-square statistic for inconsistency.

We quantified the effect and assessed the potential heterogeneity of effect between studies (with Cochran’s Q test and the *I^2^* metric, Table 3 and Figure 2, Figure 3, and Figure 4). For rs3754219, significant heterogeneity of effect between studies was demonstrated, not only for all AMD cases combined (p=0.006; *I*^2^=70%) but also for CNV (p=0.02; *I*^2^=65%) and late AMD cases (p=0.002; *I*^2^=73%). Furthermore, moderate (not significant) heterogeneity was seen for the GA and CNV + mixed cases, respectively. No heterogeneity was found for the early and mixed AMD cases (Table 3). For the groups with *I*^2^ values >25%, we used the random effects model (the DerSimonian and Laird method) to calculate the pooled OR; otherwise, in absence of heterogeneity (*I*^2^ statistic <25%), we used the fixed effects model (the Mantel-Haenszel method). The summary OR indicated that no significant association between rs3754219 and (all clinical subtypes of) AMD was found over all populations (Figure 2). Similarly, meta-analysis for rs4660687 showed significant heterogeneity (p=0.02; *I^2^*=64%) for all AMD cases. Moderate (but not significant) heterogeneity was seen for the AMD subtypes with the exception of the GA, CNV, and CNV+ mixed cases where no or low heterogeneity was detected (Table 3). Using a random or fixed effects model (depending on the amount of heterogeneity), we found no association between rs4660687 and (all clinical subtypes of) AMD over all populations (Figure 3). Also for rs841853, the between-study heterogeneity was significant; not only for all AMD cases combined (p=0.008; *I*^2^=68%) but also for CNV (p=0.0005; *I*^2^=80%) and late AMD cases compared to the controls (p=0.002; *I*^2^=74%). We saw moderate (not significant) heterogeneity for CNV+ mixed cases and saw no or low heterogeneity for the early, GA, and mixed cases (Table 3). Using a random or fixed effects model (depending on the amount of heterogeneity), the summary OR indicated that no significant associations were present between rs841853 and (all clinical subtypes of) AMD (Figure 4).

**Table 3:** Test for heterogeneity of effect between different populations for *SLC2A1* SNPs rs3754219, rs4660687 and rs841853.

** **	rs3754219				rs4660687				rs841853			
**Controls versus**	**χ^2^**	**df**	**p-value**	**I^2^**	**χ^2^**	**df**	**p-value**	**I^2^**	**χ^2^**	**df**	**p-value**	**I^2^**
All AMD cases combined	16.4	5	0.006	70%	14	5	0.02	64%	15.74	5	0.008	68%
Early	4.2	5	0.52	0%	8.79	5	0.12	43%	4.27	5	0.51	0%
GA	7.57	4	0.11	47%	1.96	4	0.74	0%	4.49	4	0.34	11%
CNV	11.59	4	0.02	65%	2.84	4	0.59	0%	20.06	4	0.0005	80%
MIXED	2.24	3	0.52	0%	4.92	3	0.18	39%	3.83	3	0.28	22%
CNV + MIXED	5.83	4	0.21	31%	4.61	4	0.33	13%	5.73	4	0.22	30%
Late (CNV+ GA+ MIXED)	18.77	5	0.002	73%	7.75	5	0.17	36%	19	5	0.002	74%

Heterogeneity between studies was tested using Cochran's Q statistic and the I-square (I^2^) statistic for inconsistency. Abbreviations: AMD=age-related macular degeneration; GA=geographic athrophy; CNV=choroidal neovascularization; Mixed=combination of GA + CNV; χ^2^=Chi square; df=degrees of freedom.

### Genetic heterogeneity between study populations

To explore the genetic contribution to the heterogeneity of effect between the populations, we assessed the differences in MAF and genotype frequencies between the study populations (Table 2 and Appendix 2). For rs3754219, we observed MAFs in the control populations ranging from 41% (Columbia University) to 52% (AMRO-NL). For the cases, we observed rs3754219 MAFs varying from 41% (AMRO-NL) to 46% (Franconian AMD study). For rs4660687, we observed MAFs in the control populations ranging from 34% (AMRO-NL) to 43% (AREDS). For the cases, the rs4660687 MAFs were nearly similar (41%–43%) with the exception of the Franconian AMD study (MAF=37%). For rs841853, the MAF ranged from 25% (AMRO-NL) to 33% (the Franconian AMD study) in the control groups. For the cases, we observed rs841853 MAFs varying from 30% (Iowa) to 35% (AMRO-NL). Subsequently, we compared the genotype frequencies of the controls between study populations for all three SNPs using the Pearson χ^2^ test: for rs3754219 and rs841853, we found significant differences with χ^2^ overall p values of 0.027 and 0.024, respectively (Table 4). We found no significant differences in genotype frequencies between the control groups for rs4660687. Next, we compared the genotype frequencies for all populations’ cases: the rs3754219 genotype frequencies of all AMD cases combined or AMD clinical subtypes of the six study populations did not show significant differences. For rs4660687, we found a significant (χ^2^ overall p value=0.002) difference in the genotype frequencies of all AMD cases and the CNV+ mixed cases (χ^2^ overall p value=0.049). For rs841853, we found significant differences in the genotype frequencies for the CNV subtype (p value=0.024; Table 4). Detailed results of the individual analysis are presented in Appendix 6.

**Table 4:** Comparison of genotype frequencies of the controls and AMD (subgroup) cases between all study populations

** **	rs3754219			rs4660687			rs841853		
** **	**χ^2^**	**df**	**Overall p-value**	**χ^2^**	**df**	**Overall p-value**	**χ^2^**	**df**	**Overall p-value**
Controls	20.26	10	0.027	14.89	10	0.136	20.60	10	0.024
All AMD	8.49	10	0.581	28.30	10	0.002	8.37	10	0.593
Early	6.81	10	0.743	8.42	10	0.588	5.13	10	0.882
GA	6.05	8	0.641	4.21	8	0.838	7.90	8	0.443
CNV	10.97	8	0.204	12.53	8	0.129	17.60	8	0.024
CNV + mixed	2.46	6	0.873	10.75	6	0.097	3.90	6	0.691
Late AMD	8.72	10	0.559	16.73	10	0.081	17.74	10	0.059
Mixed AMD	5.63	8	0.689	15.59	8	0.049	6.99	8	0.538

Genotype frequencies were compared by using the Pearson χ^2^ test. Abbreviations: AMD=age-related macular degeneration; GA=geographic athrophy; CNV=choroidal neovascularization; Mixed=combination of GA+CNV; χ^2^=Chi square; df=degrees of freedom.

## Discussion

Three out of 22 *SLC2A1* SNPs tested showed a significant allelic and genotypic association with AMD in the AMRO-NL discovery cohort. Replication of these three SNPs (rs3754219, rs4660687, and rs841853) in five independent cohorts yielded inconsistent association results. Meta-analysis revealed no overall association between *SLC2A1* and AMD. For all three SNPs, we observed significant heterogeneity of effect and high inconsistency across the study populations. Using a random or fixed effect model, we calculated pooled ORs, which were not significant for any of the SNPs in any of the studies. Such findings are not unique: one of the greatest challenges in interpreting genetic association studies is the lack of consistent reproducibility [48]. Hirschhorn and colleagues (2002) reviewed more than 600 positive associations between common gene variants and disease and found that the majority is not robust. Of the 166 presumed associations, which were studied three or more times, only six were consistently replicated [49]. Similarly, a meta-analysis of 301 published studies covering 25 different reported associations showed that less than half of the associations were consistent across the different study populations [50]. For AMD, at least two recent studies highlight the lack of consistent replication of association across large population studies: initial associations found between AMD and SNPs in the Toll-like receptor 3 [51] and the Serpin Peptidase Inhibitor, Clade G, member 1 (*SERPING1*) genes [52] could not be replicated independently [47,53]. For *SLC2A1* SNP rs841853 and diabetic nephropathy, a similar inconsistent association result across large study populations was reported [54].

The *SLC2A1* SNPs rs841853, rs3754219, and rs4660687 do not reside within known regulatory region(s) of the *SLC2A1* gene (i.e., promoter, enhancers, and silencer elements). We entered these SNPs into the SNPExpress database to find out that these SNPs apparently indeed do not affect gene expression. Obviously, the possibility exists that one or more of the SNPs tag a true causal variant in or adjacent to the *SLC2A1* gene that determines the possible genetic susceptibility to AMD. Interestingly, Tao and coworkers (1995) examined whether the association between the rs841853 SNP and non-insulin-dependent diabetes mellitus could be due to other causal variants in LD with rs841853. Initially, such a variant was not found [55]. However, after seven years, Ng et al. (2002) found a new putative causal variant in LD with rs841853 in an insulin-responsive enhancer element, potentially regulating *SLC2A1* gene expression and/or function [56].

One of our study limitations is that we selected for common *SLC2A1* SNPs, with MAFs>10%. Consequently, we could have missed variants with MAFs<10% and outside the coding region that might influence the disease phenotype. In addition, because our initial association values found in the AMRO-NL study passed only two out of three significance levels used (see Material and Methods), there was, initially, a somewhat increased possibility of false-positive association(s). Furthermore, as we and others have discussed extensively elsewhere [36,57], inconsistent replication in genetic-association studies can be caused by numerous factors, including chance, variation in study design, phenotypic AMD (grading) differences, and genotype errors [58]. Indeed, the effect of these factors is stronger in small cohorts, such as defined by clinical subtypes of AMD. These factors may have played a role in our study [36]. In addition, genetic heterogeneity between populations may significantly affect the outcome of association studies [57]. It may be not coincidental that we found positive replication for *SLC2A1* SNP rs4660687 in the early cases of the Dutch populations (the AMRO-NL and the Rotterdam Study cohorts), where the allele and genotype distributions are more or less similar, but not in other populations.

Interestingly, we found opposite associations for the three *SLC2A1* SNPs (rs3754219, rs4660687, and rs841853) in two highly comparable populations. A similar “flip-flop” association was previously found by Lin et al. in two different ethnic populations [59]. The source of this opposite effect in our studies is not clear, and remains to be elucidated.

To assess the contribution of “the genetic factor” in detail, we compared the genotype frequencies of all study populations in relation to the heterogeneity of effect. The Pearson χ^2^ test showed that genotype frequencies for *SLC2A1* rs3754219 and rs841853 were significantly different for the controls of the six individual populations. For rs4660687, we observed significant differences in genotype distributions for the cases. For several *SLC2A1* SNPs, including rs841853, genuine genetic differences between populations exist [27,28,60–63]. The fact that we found significant genetic heterogeneity for three *SLC2A1* SNPs in highly comparable (Caucasian) populations strongly suggests that the genetic contribution to the heterogeneity of effect observed in our study is substantial.

In summary, in this study we found no consistent significant association between three *SLC2A1* SNPs (rs3754219, rs4660687, rs841853) and AMD. The heterogeneous findings on these three *SLC2A1* SNPs provide further evidence for population-dependent genetic risk heterogeneity in AMD [47,51–53]. Further studies in other, ethnically similar and diverse populations are needed to falsify, confirm, or substantiate the potential role of *SLC2A1* in AMD.

## Appendix 1. Age-related macular degeneration classification per study sample.

AMD=age-related macular degeneration; GA=geographic athrophy; CNV=choroidal neovascularization; Mixed AMD=combination of GA + CNV. To access the data, click or select the words “Appendix 1.” This will initiate the download of a compressed (pdf) archive that contains the file.

## Appendix 2. Risk of AMD for SLC2A1 SNPs rs3754219, rs4660687 and rs841853 in five replication populations.

AMD=age-related macular degeneration; CI=confidence interval; OR=odds ratio; SNP=single nucleotide polymorphism; m.a.f=minor allele frequency. “A” indicates common allele, “a” minor allele. Percentages not always add up to 100% because of conversion to whole numbers. ORs were estimated by logistic regression. Adjustment for age and gender only in the Rotterdam Study. To access the data, click or select the words “Appendix 2.” This will initiate the download of a compressed (pdf) archive that contains the file.

## Appendix 3. Risk of AMD (subgroup) cases for SLC2A1 SNP rs3754219 in five replication populations.

AMD=age-related macular degeneration; CI=confidence interval; OR=odds ratio; SNP=single nucleotide polymorphism; m.a.f=minor allele frequency; GA=geographic athrophy; CNV=choroidal neovascularization; Mixed AMD=combination of GA + CNV; N.p=not performed. “A” indicates common allele, “a” minor allele. Percentages not always 100% because of conversion to whole numbers. ORs are estimated with logistic regression analysis. Adjustment for age and gender only in the Rotterdam study. To access the data, click or select the words “Appendix 3.” This will initiate the download of a compressed (pdf) archive that contains the file.

## Appendix 4. Risk of AMD (subgroup) cases for SLC2A1 SNP rs4660687 in five replication populations.

AMD=age-related macular degeneration; CI=confidence interval; OR=odds ratio; SNP=single nucleotide polymorphism; m.a.f=minor allele frequency; GA=geographic athrophy; CNV=choroidal neovascularization; Mixed AMD=combination of GA + CNV; N.p=not performed. “A” indicates common allele, “a” minor allele. Percentages not always 100% because of conversion to whole numbers. ORs are estimated with logistic regression analysis. Adjustment for age and gender only in the Rotterdam study. To access the data, click or select the words “Appendix 4.” This will initiate the download of a compressed (pdf) archive that contains the file.

## Appendix 5. Risk of AMD (subgroup) cases for *SLC2A1* SNP rs841853 in five replication populations.

AMD=age-related macular degeneration; CI=confidence interval; OR=odds ratio; SNP=single nucleotide polymorphism; m.a.f=minor allele frequency; GA=geographic athrophy; CNV=choroidal neovascularization; Mixed AMD=combination of GA + CNV; N.p=not performed. “A” indicates common allele, “a” minor allele. Percentages not always 100% because of conversion to whole numbers. ORs are estimated with logistic regression analysis. Adjustment for age and gender only in the Rotterdam study. To access the data, click or select the words “Appendix 5.” This will initiate the download of a compressed (pdf) archive that contains the file.

## Appendix 6. Comparison of genotype frequencies of the controls and AMD (subgroup) cases between all study populations.

Genotype frequencies were compared by using the Pearson χ^2^ test. Abbreviations: AMD=age-related macular degeneration; GA=geographic athrophy; CNV=choroidal neovascularization; Mixed=combination of GA+CNV; χ^2^=Chi square; df=degrees of freedom. To access the data, click or select the words “Appendix 6.” This will initiate the download of a compressed (pdf) archive that contains the file.
